# 2379. An Urban Detroit Clinic’s Experience in Bivalent COVID-19 Booster Uptake in People Living with HIV

**DOI:** 10.1093/ofid/ofad500.2000

**Published:** 2023-11-27

**Authors:** Jahanavi M Ramakrishna, Abdullah Yesilyaprak, Lea M Monday, Gretchen Snoeyenbos Newman

**Affiliations:** Wayne State University, Rochester, Michigan; Wayne State University School of Medicine, Rochester, Michigan; Wayne state University School of Medicine, Detroit, Michigan; Wayne State University School of Medicine, Rochester, Michigan

## Abstract

**Background:**

Both HIV and COVID-19 disproportionately affect vulnerable communities. People living with HIV (PLWH) are a priority population for COVID-19 vaccination. Bivalent COVID-19 boosters (BCB) were granted emergency use authorization on August 31, 2022. We assessed onsite BCB uptake amongst PLWH enrolled at our HIV clinic in Detroit, Michigan.

**Methods:**

HIV care data was obtained from CareWare collected as part of Ryan White program quality initiatives. BCB (Pfizer/BioNTech and Moderna) administration data from September 1, 2022 to March 20, 2023 was pulled using Current Procedural Terminology codes from our Athena electronic medical record. We excluded patients < 18 years old. A deterministic match was done using name and date of birth. Of 2268 PLWH, 1,921 (84.7%) had complete care data. The outcome of interest was the percentage of patients who received the BCB. Covariates examined were gender, age (years), race, CDC transmission risk factor, year of HIV diagnosis, insurance type, HIV viral load measurement after Sep. 1, 2022, and most recent HIV viral load value (< or > 200 copies/mL). Bivariate analysis was done using Wilcoxon rank sum test and Pearson's Chi-squared test. Statistical significance was defined as *P* < 0.05. We used R 4.3.0 for all analyses.

**Results:**

Of 1,921 PLHW, 519 (27%) received the BCB. Compared to PLWH 18-29, those 50 years and older were more likely to receive the BCB (*P* < 0.001). Although not statistically significant, Black PLWH (26%) were less likely to have been vaccinated with the BCB compared to white PLWH (32%, *P* < 0.30). BCB uptake was higher in PLWH diagnosed with HIV prior to 2010 (*P* < 0.001). PLWH enrolled in Medicare were more likely to receive the BCB than those with other or no insurance (*P* < 0.001). No statistically significant trends were found based on CDC HIV transmission risk factor. Only 5% of PLWH who underwent HIV viral load testing before Sep. 1, 2022 received the BCB compared to 33% with testing after Sep. 1, 2022 (*P* < 0.001). Patients with viral load < 200 copies/mL (28%) had a higher BCB vaccination rate compared to those with viral load > 200 copies/mL (16%, *P* < 0.001).

**Figure:**
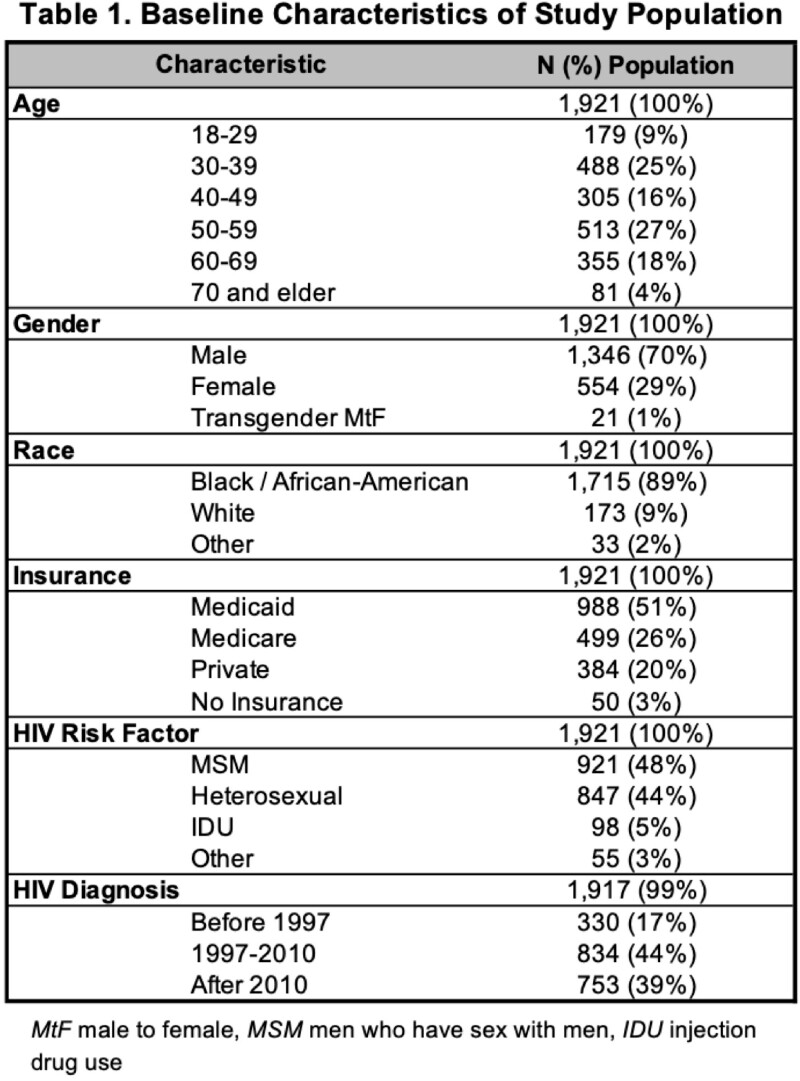

**Figure:**
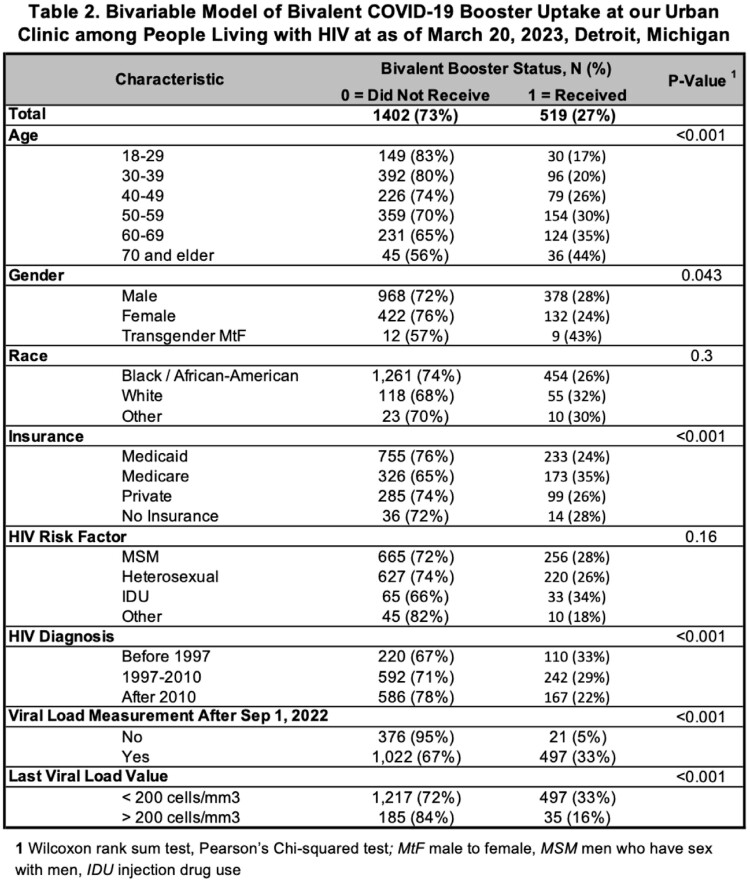

**Conclusion:**

Vaccine surveillance studies such as this one are critical to equitable vaccine distribution to PLWH by identifying subpopulations that may benefit from more intensive outreach.

**Disclosures:**

**All Authors**: No reported disclosures

